# Supplementary material for: YtfK activates the stringent response by triggering the alarmone synthetase SpoT in Escherichia coli
Source: Nat Commun. 2019 Dec 17;10:5763. doi: 10.1038/s41467-019-13764-4 (PMC6917717; doi:10.1038/s41467-019-13764-4)
Supplement: Supplementary file 1 — Supplementary Information [file 41467_2019_13764_MOESM1_ESM.pdf]

Supplementary Information to

YtfK activates the stringent response by triggering the alarmone synthetase SpoT in *Escherichia coli*

Elsa Germain[1]*, Paul Guiraud[1], Deborah Byrne[2], Badreddine Douzi[1†], Meriem Djendli[1], and Etienne Maisonneuve[1]*

[1] Laboratoire de Chimie Bactérienne, Institut de Microbiologie de la Méditerranée, CNRS-Aix Marseille Univ (UMR7283), Marseille, France

[2] Protein Expression Facility, Institut de Microbiologie de la Méditerranée, CNRS-Aix Marseille Univ, Marseille, France

[†] Present address : Université de Lorraine, Inra, DynAMic, F-54000 Nancy, France

***e-mail: egermain@imm.cnrs.fr; emaisonneuve@imm.cnrs.fr**

Supplementary Table 1: Strains used in this work

**Strains**	**Genotype**	**Source**
BTH101	F- cya-99 araD139 galE15 galK16 rpsL(Str^R) hsdR2 mcrA1 mcrB1 relA1	(Karimova et al., 1998)[1]
XL1red	endA1 gyrA96 thi-1 hsdR17 supE44 relA1 lac mutD5 mutS mutT Tn10 (Tetr) a	Agilent Technologies
BL21 (DE3)	*E. coli* B F$^-$ *ompT gal*	NEB
DY331	W3110 ΔlacU169 gal490 pglΔ8 [λ cI857 Δ(cro-bioA)] Δ(srlA-recA)301::Tn10	(Thomason et al ., 2007)[2]
MG1655	Wild-type *E.coli*	
ΔrelA	MG1655 *relA::FRT*	P1 from KEIO collection. (Resistance cassette has been flipped out)[3].
ΔrelA spoT	MG1655 *relA251::aphA spoT207*::*cat*	Sequential P1 transduction from CF1693[4] in MG1655
ΔytfK	MG1655 *ytfK*::FRT	P1 from KEIO collection (Resistance cassette has been flipped out)[3].
ΔrelA ytfK	MG1655 *relA*::FRT *ytfK*::FRT	P1 from KEIO collection (Resistance cassette has been flipped out)[3].
ytfK-mNeonGreen	MG1655 *ytfK-mNeonGreen*	This study

Supplementary Table 2: List of active residues used as input for Spot/YtfK docking simulation

Protein	***Active residues number***
SpoT	3,5,9,10,11,12,13,14,15,16,17,19,21,22,24,25,27,28,31,32,35,37,38,39,40,41,43,48,58,59,60,61,64,76,77,78,79,81,82,83, 84,85,86,87,88,95,98,101,102,104,105,108,109,110,111,112,113,117,119,121,124,128,142,146,147,149,151,152,154,156, 157,160,164,171,176,177,180,184,185,188,191,193,194,196,197,199,200,203,204,208,210,211,214,215,217,221,223,225, 226,229,230,231,232,234,237,240,248,251,252,253,255,256,257,258,260,266,268,269,270,271,275,279,282,283,285,287, 289,290,291,292,297,300,306,311,314,316,317,325,326,328,329,330,333,340,344,345,346,347,348,350,354,356,359,360, 364,365,367,369,371,373,375,376,377,378
YtfK	1,2,3,4,5,6,7,8,9,10,11,14,15,17,18,19,20,21,23,25,27,28,29,31,33,35,36,38,40,41,42, 43,44,45,46,47,48,49,50,51,53,54,55, 57,58,59,61,62,63,64,66

Supplementary Table 3: Parameters of the best five clusters generated by Haddock.

Parameters	*Cluster 8*	*Cluster 5*	*Cluster 1*	*Cluster 3*	*Cluster 6*
HADDOCK Score	132.2±13.6	148.9±18.3	158.0 +/- 7.2	160.7 +/- 7.2	163.5 +/- 10.3
Cluster size	6	7	14	8	7
RMSD from the overall lowest-energy structure	3.3 ± 0.2	5.1 ± 0.0	5.1 ± 0.0	5.8 ± 0.1	3.2 ± 0.2
Van Der Waals energy	-116.2 ± 14.3	-111.4 ± 5.0	-117.0 ± 12.2	-107.2 ± 9.2	-93.2 ± 9.4
Electrostatic energy	-529.9 ± 25.7	-499.6 ± 71.4	-531.1 ±109.8	-495.8 ± 40.7	-534.4 ± 50.7
Desolvation energy	26.9 ± 4.4	30.1 ± 11.2	49.8 ± 10.5	42.0 ± 5.8	37.4 ± 8.0
Restraints violation energy	3275.4 ± 224.19	3301.2 ± 154.38	3314.7 ± 299.51	3251.0 ± 37.48	3261.6 ± 92.25
Buried Surface Area	3974.4 ± 214.2	3686.3 ± 49.6	3783.2 ± 229.0	3585.3 ± 93.5	3475.1 ± 133.6
Z-Score	-2.2	-1.0	-0.4	-0.2	-0.0

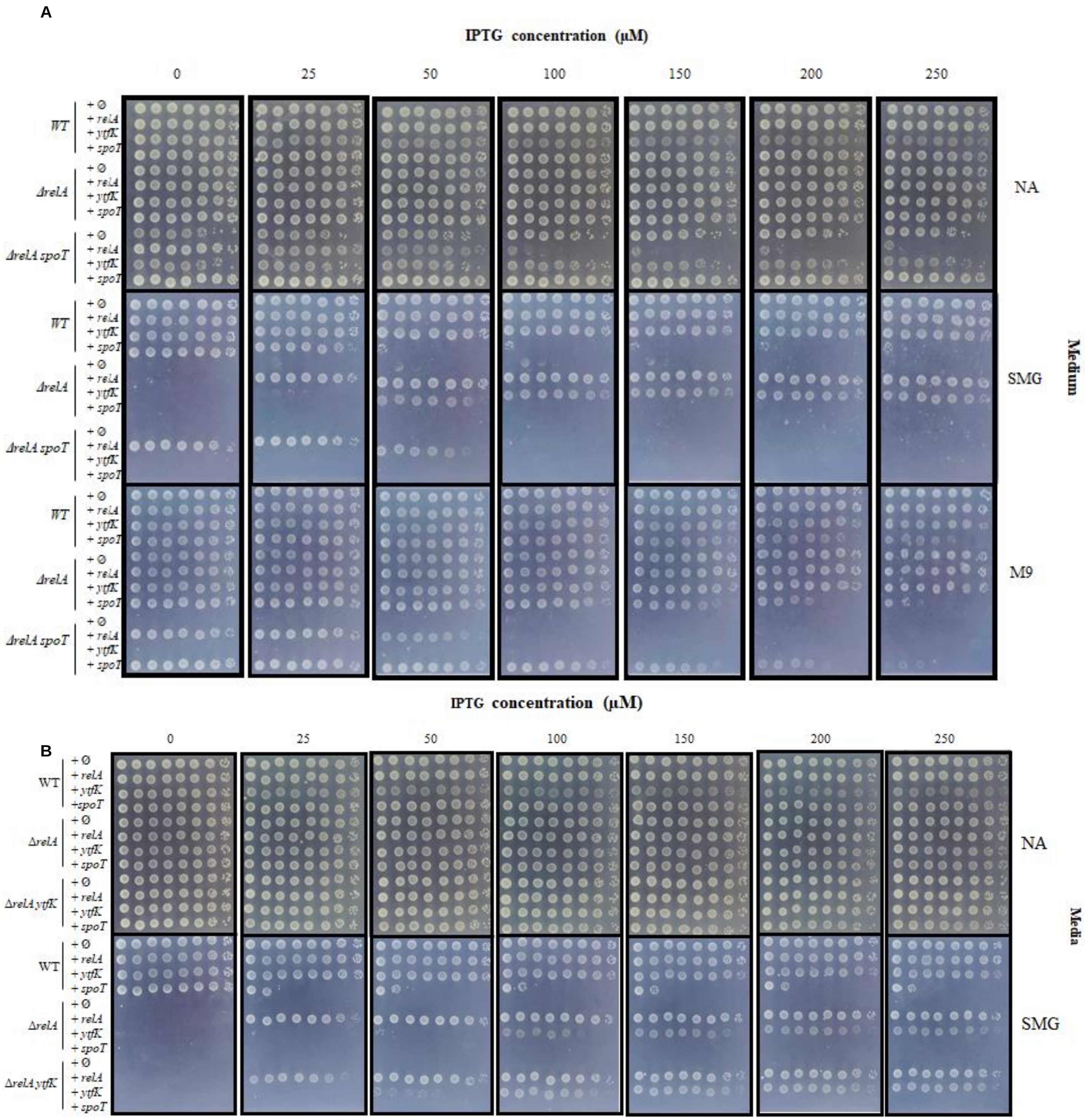

Supplementary Figure 1: (A) YtfK is not a ppGpp synthetase WT, *ΔrelA* and *ΔrelA spoT* mutants were transformed with pEG25 harboring either *relA*, *ytfK* or *spoT* under an IPTG inducible promoter. Cells were serially diluted and spotted on both nutrient agar (NA), minimal medium with an excess of Ser, Met, Gly (SMG) or minimal medium agar plates (M9) without amino acids and in presence of increasing concentrations of IPTG. (**B**) **YtfK suppresses the growth defect of a *ΔrelA ytfK* double mutant strain**. WT, *ΔrelA* and *ΔrelA ytfK* mutants were transformed with pEG25 harboring either *relA*, *ytfK* or *spoT* under an IPTG inducible promoter. Cells were serially diluted and spotted on both nutrient agar (NA) or on minimal medium with an excess of Ser, Met, Gly (SMG).

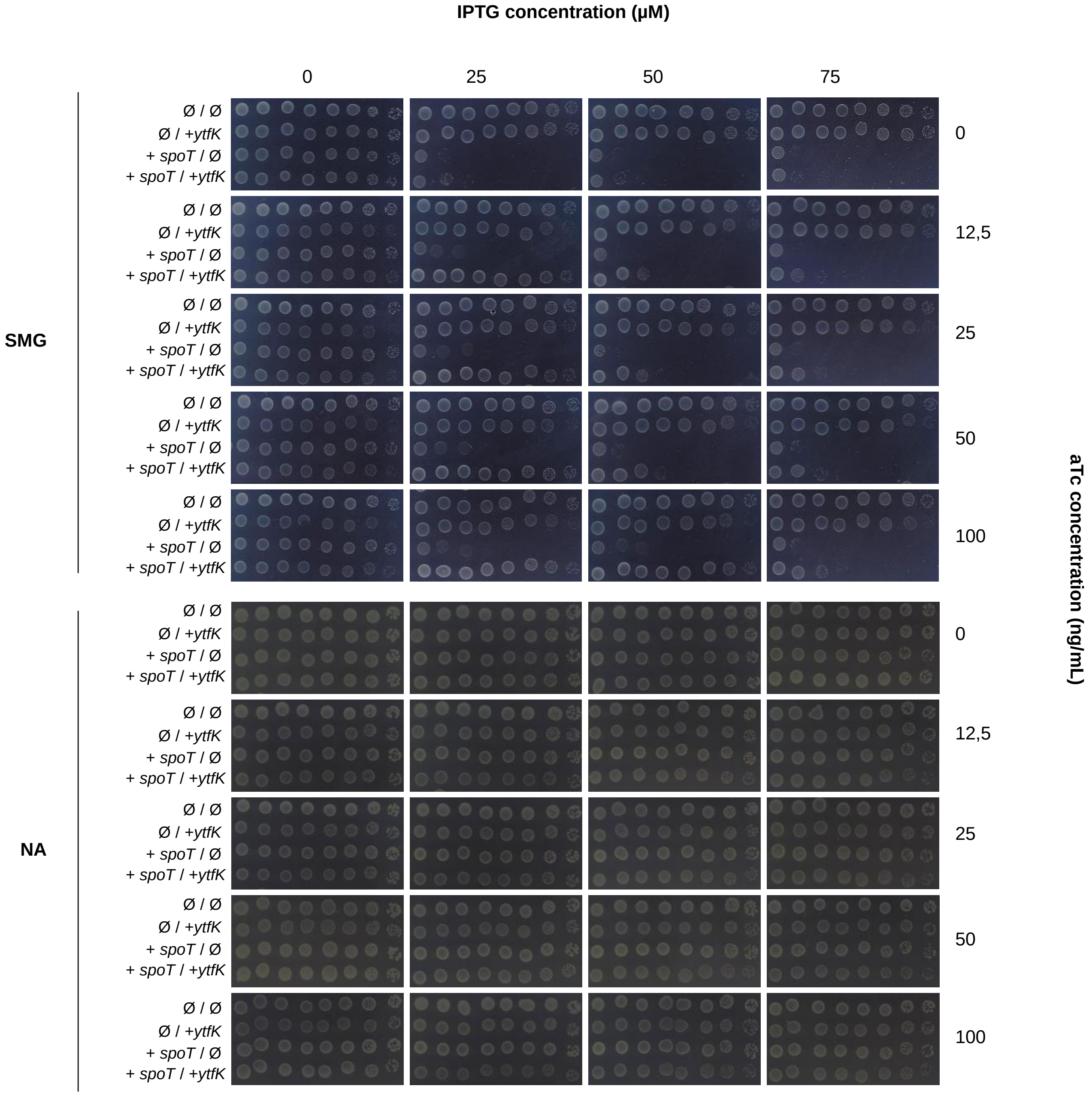

Supplementary Figure 2: The YtfK / SpoT ratio determines growth on SMG plates
WT strain was co-transformed with pEG25 or pEG25 harboring *spoT* gene under an IPTG inducible promoter and with pBbS2K or pBbS2K harboring *ytfK* gene under an anhydrotetracyclin (aTc) promoter. Cells were serial diluted and spotted both on SMG and NA with gradual concentration of IPTG (to induce *spoT*) and aTc (to induce *ytfK*). Experiments have been repeated 4 times with similar results.

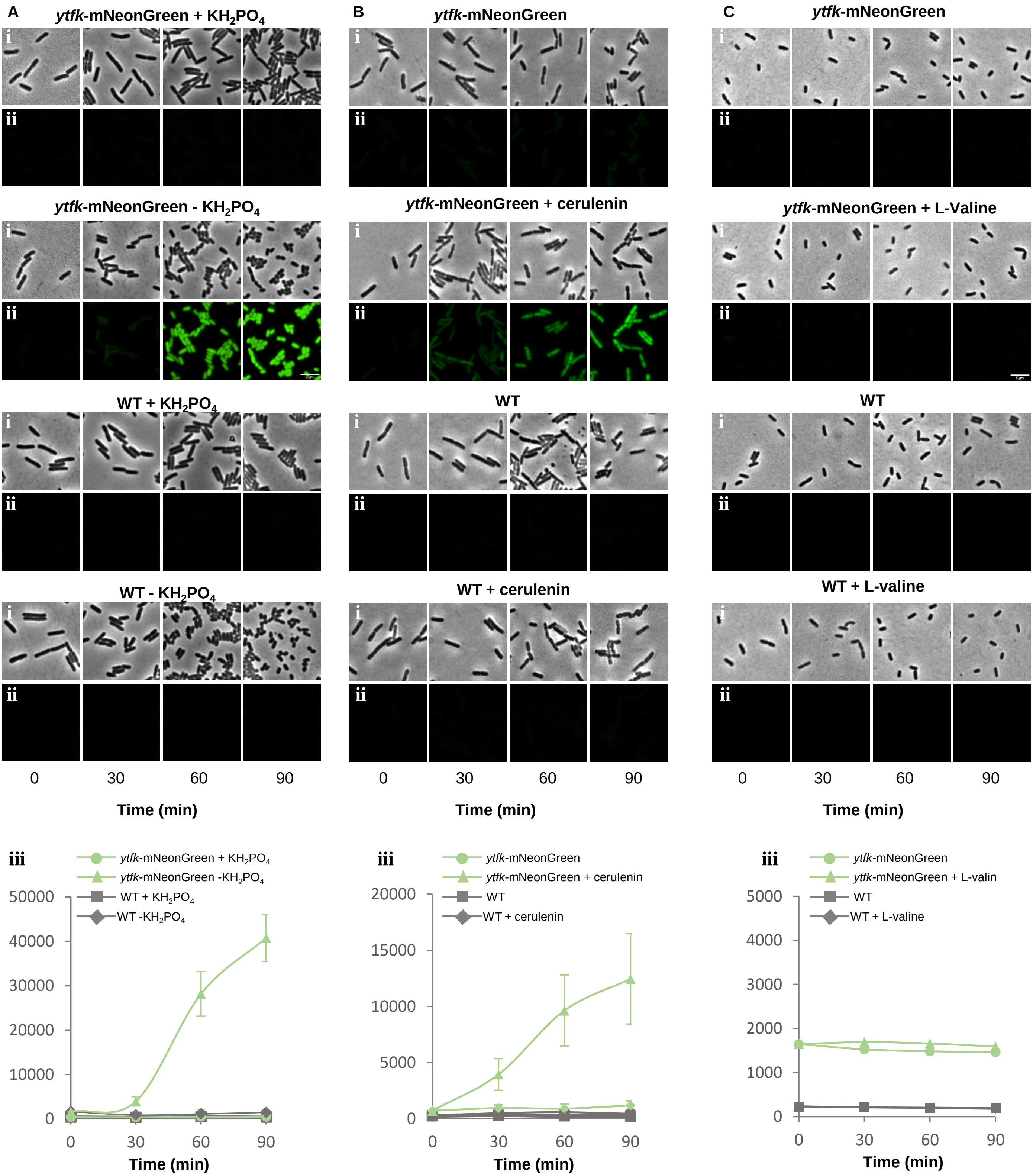

Supplementary Figure 3: Expression of *ytfK* upon phosphate, fatty acid and amino acid starvations

WT and *ytfK-mNeonGreen* strains were grown under replete and deplete conditions; Phopsphate (**A**) fatty acid (**B**) or amino acids (**C**) as described in supplementary methods. For each condition Snap-shot of cells at the indicated time is shown (i) diascopy (DIA) images (ii) fluorescence images. (iii) Fluorescence quantification. Error bars indicate the standard deviation of average fluorescence measured on at least 367 cells for each set.

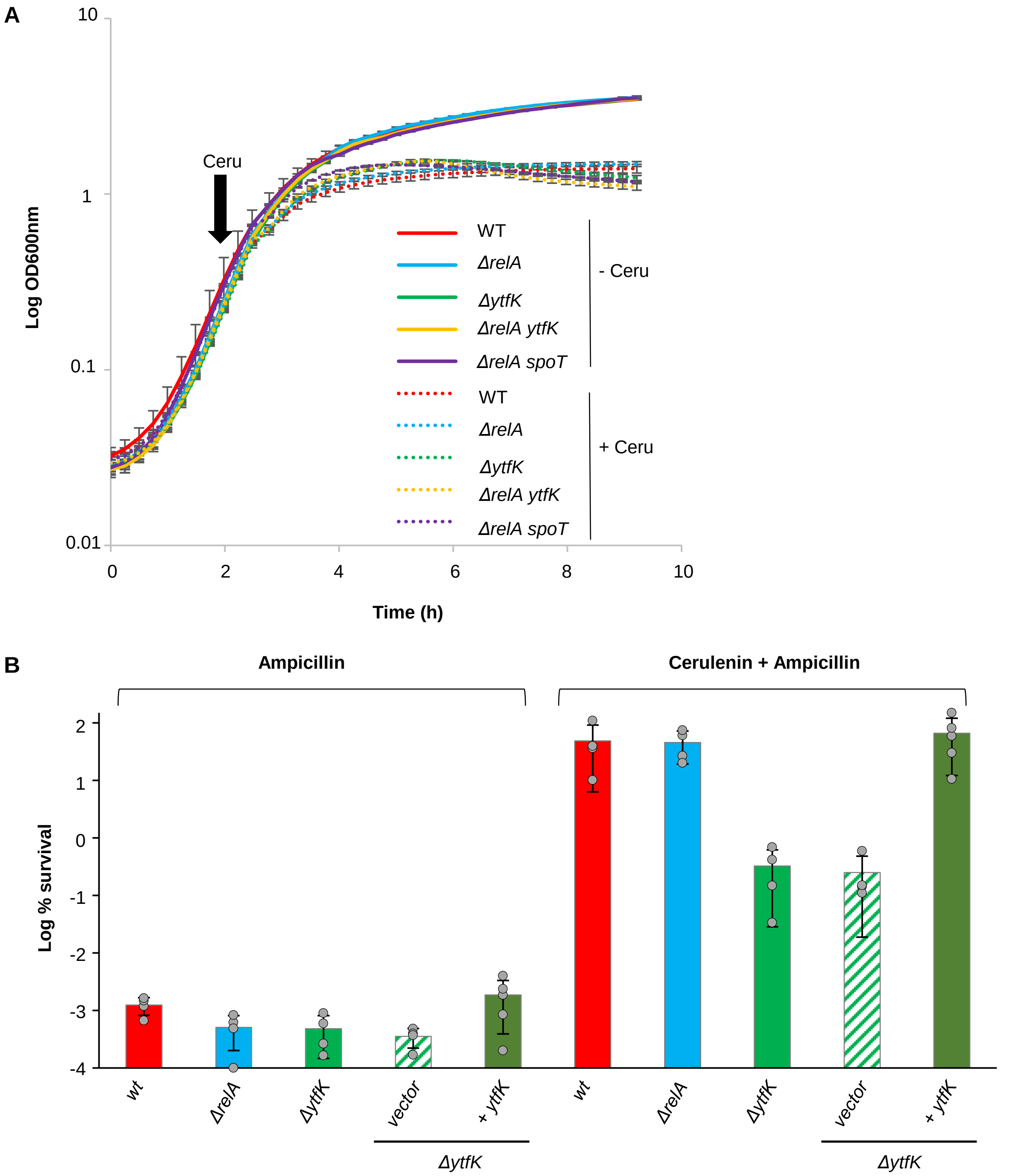

Supplementary Figure 4: YtfK contributes to antibiotic tolerance

(A) Growth curve upon cerulenin treatment. Briefly, after overnight culture, WT, *ΔrelA*, *ΔytfK*, *ΔrelA ytfK* and *ΔrelA spoT* strains cells were 1/1000 diluted in a microplate and growth was monitored at 600nm using microplate reader for 9H00. At OD_{600}:0,6, 250µg/ml of cerulenin has been added into the cultures and optical density at 600nm has been followed for 4H00. (**B**) YtfK is required for stress-induced ampicillin tolerance. Growing cells of MG1655 (WT) and isogenic deletion strains *ΔrelA*, *ΔytfK*, Δ*ytfK* harboring the pEG220 and the Δ*ytfK* harboring either the pEG220-*ytfK* (with native promoter) were exposed to 100 µg/mL of ampicillin for 4h (left panel) or exposed to the same dose of ampicillin after 30 min of cerulenin pretreatment (250 µg/mL) (right panel). Percentage of survival (log scale) after 4h is shown. Error bars indicate the SDs of averages of three independent experiments. Source data are provided as a Source Data file.

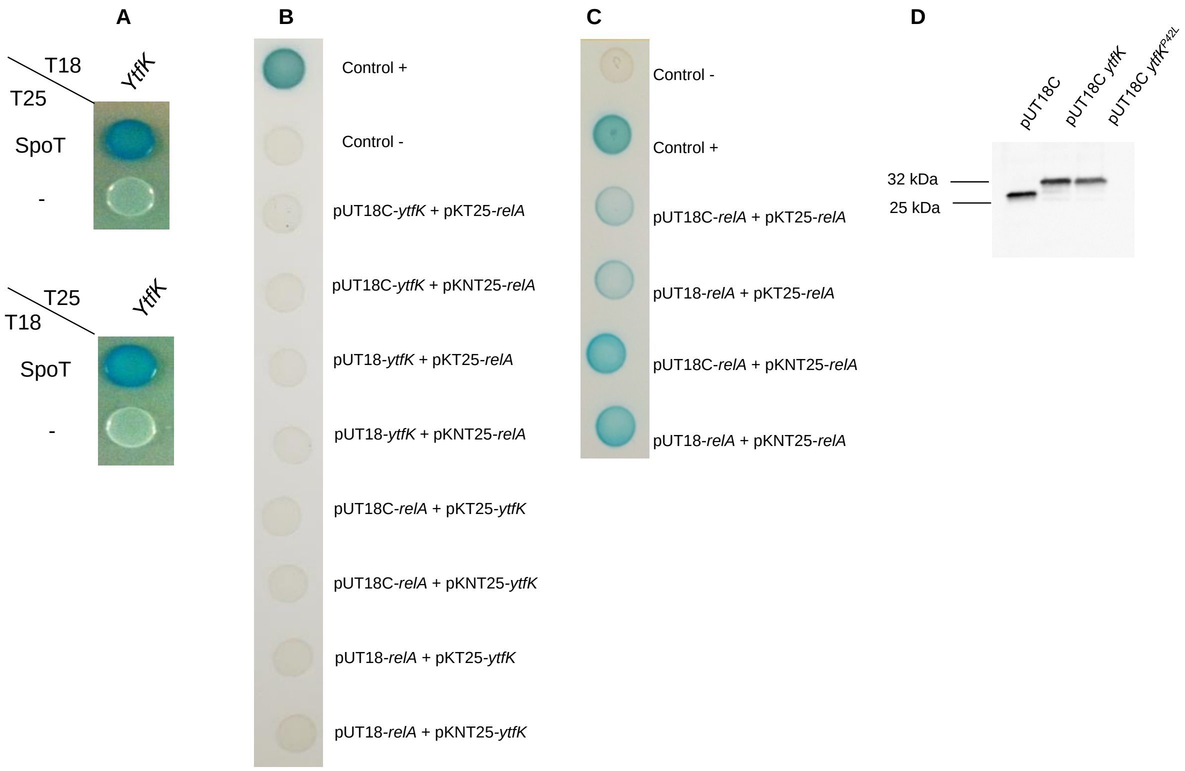

Supplementary Figure 5: Functional interaction between YtfK and SpoT

(A) Bacterial two hybrid assay with YtfK and SpoT. BTH101 cells were co-transformed with plasmids harboring T18 and T25 fusions (or empty plasmid (-)) were spotted on X-Gal agar base plates (Methods). (**B**) Bacterial two hybrid between YtfK and RelA in all combinations. (**C**) Bacterial two hybrid showing the multimerization of RelA in all combinations as control for the correct production of the different RelA fusions from the BTH system. The blue color indicates a positive interaction. (**D**) Control of the correct production of $YtfK^{P42L}$. Briefly, MG1655 strain was transformed with pUT18c plasmid harboring either *ytfK* or *ytfK*^{P42L}. After 3h00 of growth at 30°C (OD_{600} 0.5) in LB, proteins production was induced by addition of 1mM IPTG for 4H00. Sample were prepared and equal amount of total proteins were loaded per lane on SDS-PAGE. Immunoblot analysis were performed with a primary anti-cyaA antibody diluted 1/5.000 (Santa Cruz) and a secondary anti-mouse antibody diluted 1/10.000 (Sigma) and visualized using luminata crescendo HRP substrate (Millipore) and ImageQuant Las4000 (GE Healthcare). Source data are provided as a Source Data file.

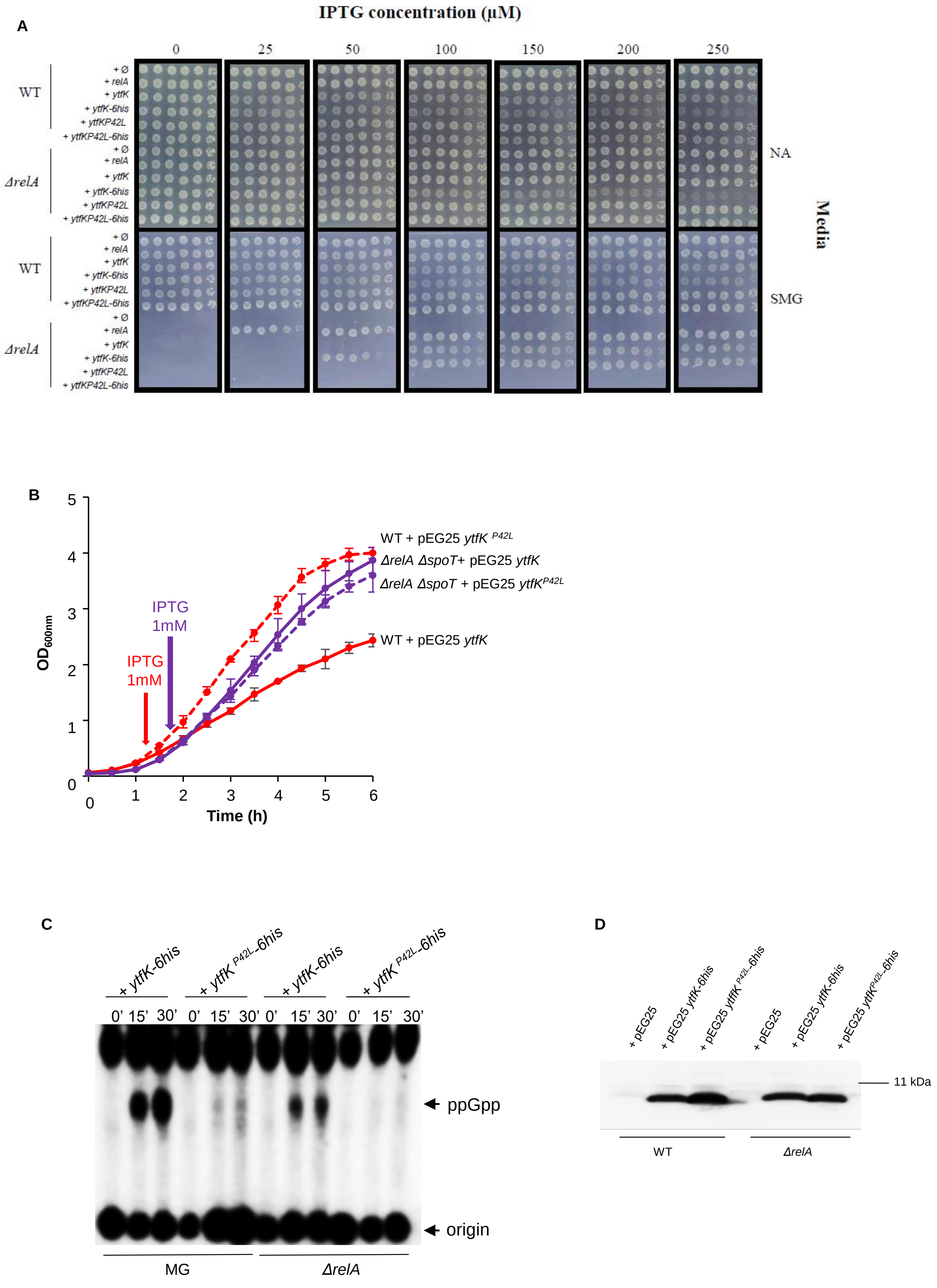

Supplementary Figure 6: Analysis of YtfK variants functionality and protein level

Supplementary Figure 6: Analysis of YtfK variants functionality and protein level
(**A**) WT and *ΔrelA* mutant were transformed with pEG25 harboring either *relA*, *ytfK*, *ytfK* ^{P42L}, *ytfK-6his, ytfK* ^{P42L}*-6his* under an IPTG inducible promoter. Cells were serial diluted and spotted both on nutrient agar (NA) and SMG plates with or without IPTG. (**B**) The intracellular level of (p)ppGpp induced by *ytfK* overexpression controls the growth rate. Growth curve of WT (red) strain or *ΔrelA ΔspoT* mutant (violet) carrying *ytfK* (solid lane) or *ytfK* ^{P42L} (dashed lane) on pEG25 in LB medium. Overnight culture were 100 times diluted and growth was monitored at 600nm. At the indicated time (70 min for the WT and 110 min for the *ΔrelA ΔspoT* mutant), 1mM of IPTG was added to induce *ytfK* expression. Error bars indicate the standard deviations of averages of three independent experiments. (**C**) *In vivo* (p)ppGpp accumulation following ectopic expression of both *ytfK-6his* and *ytfK* ^{P42L}*-6his*. WT strain or *ΔrelA* mutant carrying *ytfK-6his* or *ytfK* ^{P42L}*-6his* on pEG25 were grown exponentially in phosphate MOPS minimal medium (see methods). Samples were collected before and after *ytfK* induction (1mM IPTG) prior nucleotides extraction and were then separated by TLC. Representative autoradiograph of the TLC plates is shown. This experiment was repeated three times with similar pattern of (p)ppGpp accumulation. **(D)** Protein level of YtfK-6His and YtfK^{P24L}-6His when produced in WT and *ΔrelA* strains. Briefly, cells were grown exponentially at 37°C in LB (OD_{600} 0.5), then cells were induced with 200µM of IPTG for 1H. Sample were prepared and equal amount of total proteins were loaded per lane on SDS-PAGE. Immunoblot analysis were performed with antibody Penta-His-HRP conjugated diluted 1/10.000 (Qiagen) as described by the company and visualized by using luminata crescendo HRP substrate (Millipore) and ImageQuant Las4000 (GE Healthcare). Source data are provided as a Source Data file.

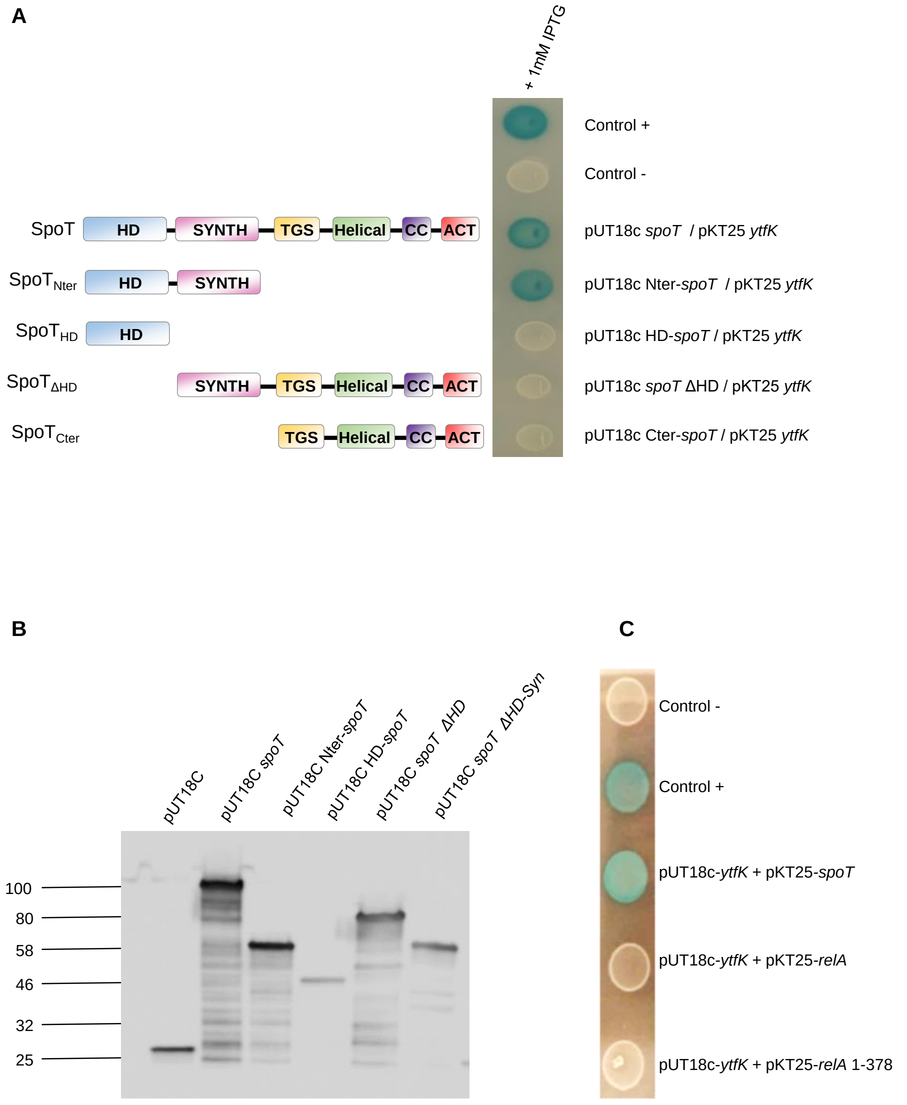

Supplementary Figure 7: Control for the production of T18-recominant SpoT and truncated versions of SpoT

(A) Bacterial two hybrid assay with SpoT and YtfK. Briefly BTH101 were co-transformed with plasmids harboring T18 and T25 fusions (or empty plasmid (-)) were spotted on X-Gal agar plates containing 1mM IPTG (Methods). **(B)** Control of the production of the all different truncated versions of SpoT encoded by pUT18C plasmid. Briefly, MG1655 strain was transformed with pUT18c harboring SpoT and truncated versions of SpoT. After 3h00 of growth at 30°C (OD_{600} 0.5) in LB, proteins production was induced by addition of 1mM IPTG for 4H00. Sample were prepared and equal amount of total proteins were loaded per lane on SDS-PAGE. Immunoblot analysis were performed with a primary anti-cyaA antibody diluted 1/5.000 (Santa Cruz) and a secondary anti-mouse antibody diluted 1/10.000 (Sigma) and visualized by using luminata crescendo HRP substrate (Millipore) and ImageQuant Las4000 (GE Healthcare). Source data are provided as a Source Data file. **(C)** Bacterial two hybrid between YtfK and RelA full length or RelA short length.

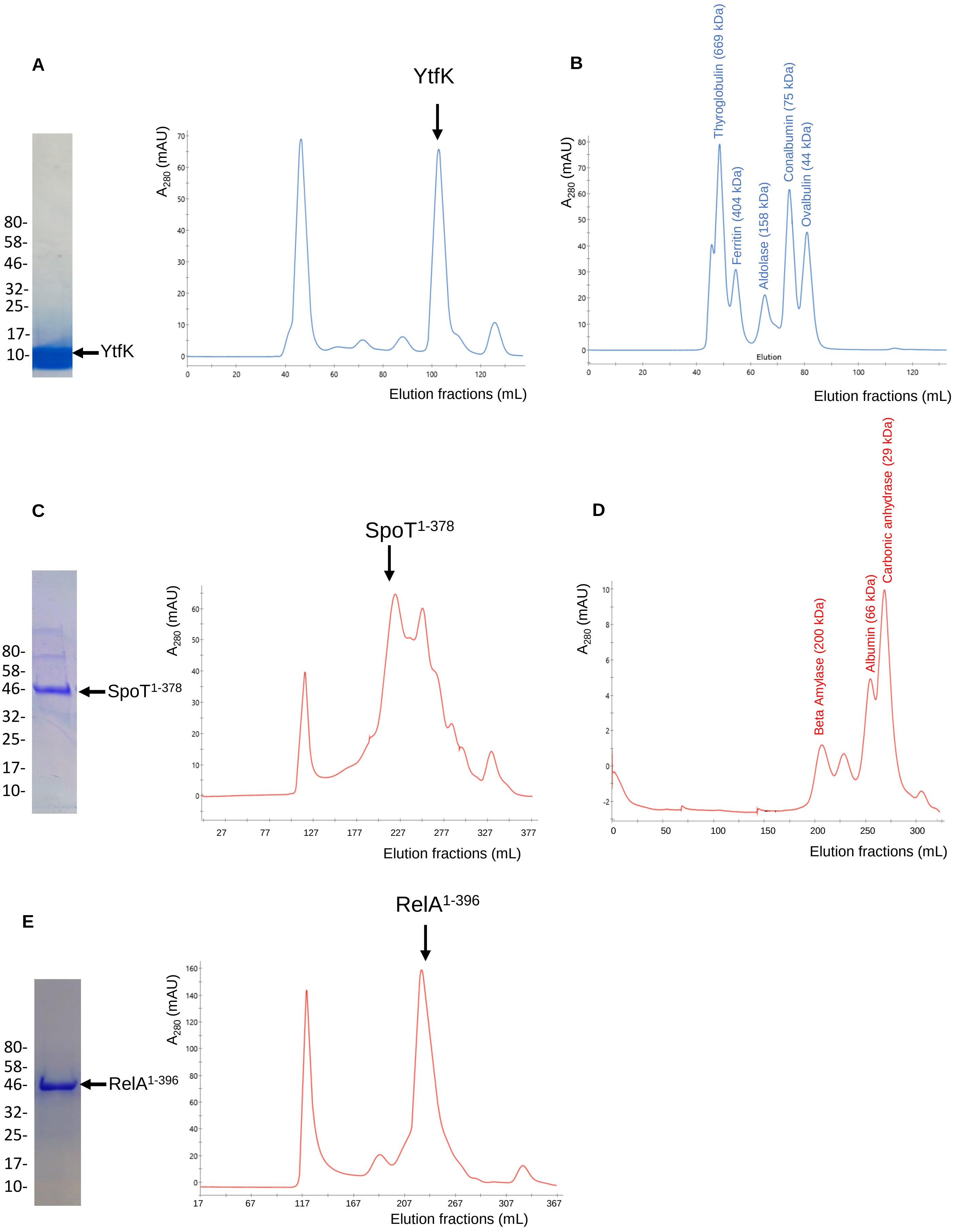

Supplementary Figure 8: YtfK SpoT$^{1-378}$ and RelA$^{1-396}$purifications on size exclusion chromatography

Supplementary Figure 8: YtfK SpoT$^{1\text{-}378}$ and RelA$^{1\text{-}396}$purifications on size exclusion chromatography

(**A**) Purification of YtfK using Size-exclusion chromatography (SEC). The elution volume (from a HiLoad 16/600 Superdex 200 column) is plotted on the x axis, and the 280-nm absorbance on the y axis. Calibration profile of the 16/600 Superdex 200 column is provided in (**B**). (**C**) Purification of SpoT$^{1\text{-}378}$ using Size-exclusion chromatography (SEC). The elution volume (from a HiLoad 26/600 Superdex 200 column) is plotted on the x axis, and the 280-nm absorbance on the y axis. Calibration profile of the 26/600 Superdex 200 column is provided in (**D**). (**E**). Purification of RelA$^{1\text{-}396}$ using Size-exclusion chromatography (SEC). The elution volume (from a HiLoad 26/600 Superdex 200 column) is plotted on the x axis, and the 280-nm absorbance on the y axis. The SEC elution fractions showed by black arrows were analyzed by 12% SDS-PAGE (Left inset). The name of the analyzed protein is indicated on the left. For SDS-PAGE molecular weight markers (in kDa) are indicated on the left.

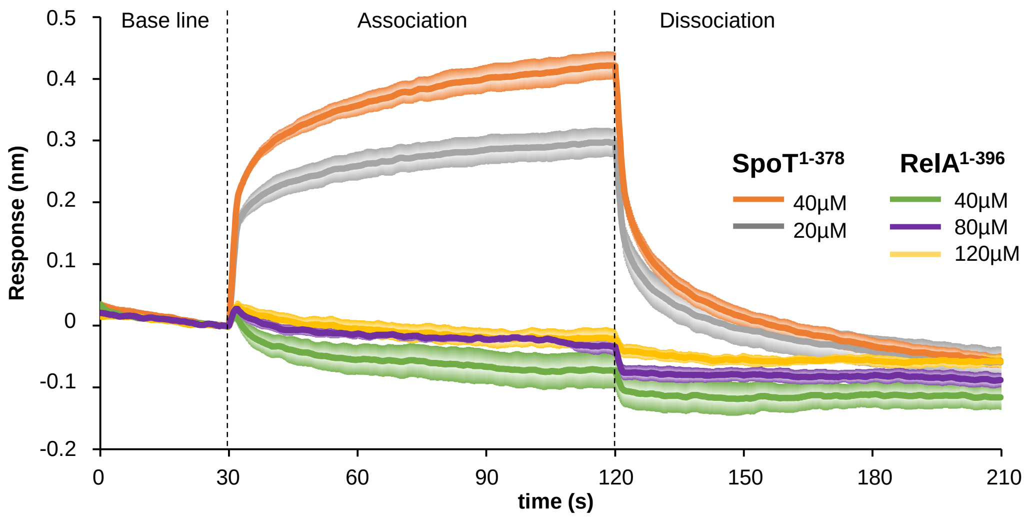

Supplementary Figure 9: YtfK does not interact with the catalytic domains of RelA *in vitro*

BioLayer interferometry assay. The graph shows the subtracted reference binding responses during the association and dissociation of SpoT or RelA on YtfK. Increasing concentrations of recombinant SpoT$^{1\text{-}378}$ (20 and 40 µM) or recombinant RelA$^{1\text{-}396}$ (40, 80 and 120µM) were bound to biotinylated YtfK (2.5µM). Error bars (presented in degraded colors) indicate the standard deviations of averages of 3 experiments. Source data are provided as a Source Data file.

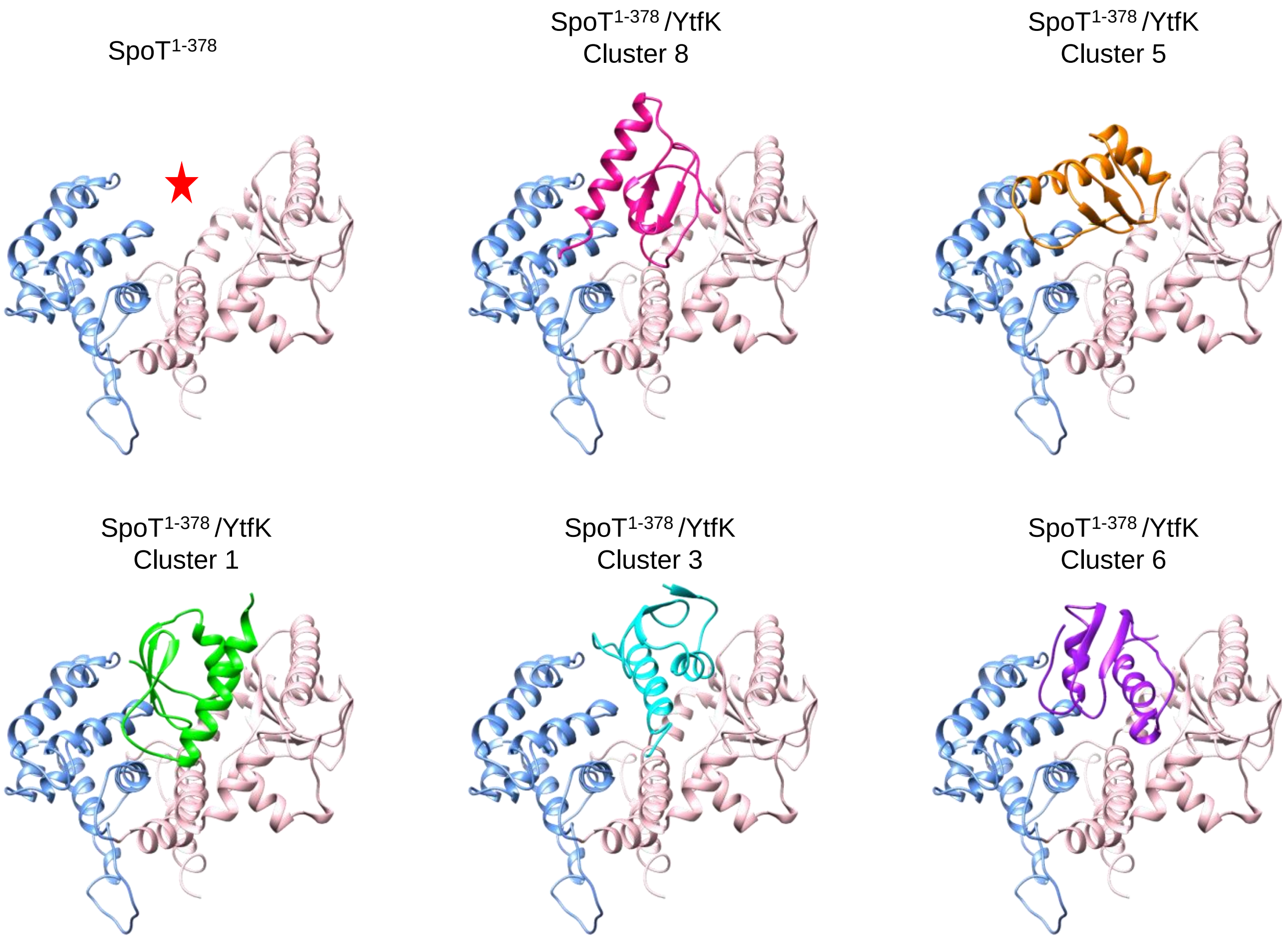

Supplementary Figure 10: **Proposed models of the *E. coli* YtfK-SpoT$^{1\text{-}378}$ assembly complex**

The modeled SpoT$^{1\text{-}378}$ is colored in blue (in marine for HD domain and light pink for SYNTH domain). The red star point to the synthetase/hydrolase interdomain contact interface.

The best fives simulated YtfK-SpoT$^{1\text{-}378}$ contact interface are presented with the modeled *E.coli* YtfK appearing in pink (cluster 8), orange (cluster 5), green (cluster 1), cyan (cluster 3), and violet (cluster 6).

Supplementary references

1. Karimova, G., Pidoux, J., Ullmann, A. & Ladant, D. A bacterial two-hybrid system based on a reconstituted signal transduction pathway. *Proc. Natl. Acad. Sci. U. S. A.* **95**, 5752–5756 (1998).

2. Thomason, L. C., Costantino, N., Shaw, D. V. & Court, D. L. Multicopy plasmid modification with phage λ Red recombineering. *Plasmid* (2007). doi:10.1016/j.plasmid.2007.03.001

3. Baba, T. *et al.* Construction of Escherichia coli K-12 in-frame, single-gene knockout mutants: The Keio collection. *Mol. Syst. Biol.* (2006). doi:10.1038/msb4100050

4. Xiao, H. *et al.* Residual guanosine 3',5'-bispyrophosphate synthetic activity of relA null mutants can be eliminated by spoT null mutations. *J. Biol. Chem.* (1991).

5. Blank, K., Hensel, M. & Gerlach, R. G. Rapid and highly efficient method for scarless mutagenesis within the salmonella enterica chromosome. *PLoS One* (2011). doi:10.1371/journal.pone.0015763

6. Datsenko, K. A. & Wanner, B. L. One-step inactivation of chromosomal genes in Escherichia coli K-12 using PCR products. *Proc. Natl. Acad. Sci. U. S. A.* (2000). doi:10.1073/pnas.120163297

7. Lee, T. S. *et al.* BglBrick vectors and datasheets: A synthetic biology platform for gene expression. *J. Biol. Eng.* (2011). doi:10.1186/1754-1611-5-12

8. Gotfredsen, M. & Gerdes, K. The Escherichia coli relBE genes belong to a new toxin-antitoxin gene family. *Mol. Microbiol.* (1998). doi:10.1046/j.1365-2958.1998.00993.x
