# Supplementary material for: YtfK activates the stringent response by triggering the alarmone synthetase SpoT in Escherichia coli
Source: Nat Commun. 2019 Dec 17;10:5763. doi: 10.1038/s41467-019-13764-4 (PMC6917717; doi:10.1038/s41467-019-13764-4)
Supplement: Supplementary file 3 — Description of Additional Supplementary Files [file 41467_2019_13764_MOESM3_ESM.pdf]

Description of Additional Supplementary Files

File Name: Supplementary Data 1

Description: List and description of plasmids used in this work.

File Name: Supplementary Data 2

Description: List of DNA oligonucleotides used in this work.
