# Supplementary material for: YtfK activates the stringent response by triggering the alarmone synthetase SpoT in Escherichia coli
Source: Nat Commun. 2019 Dec 17;10:5763. doi: 10.1038/s41467-019-13764-4 (PMC6917717; doi:10.1038/s41467-019-13764-4)
Supplement: Supplementary file 6 — Reporting Summary [file 41467_2019_13764_MOESM6_ESM.pdf]

nature research

Corresponding author(s): Etienne Maisonneuve

Reporting Summary

Nature Research wishes to improve the reproducibility of the work that we publish. This form provides structure for consistency and transparency in reporting. For further information on Nature Research policies, see Authors & Referees and the Editorial Policy Checklist.

Statistical parameters

When statistical analyses are reported, confirm that the following items are present in the relevant location (e.g. figure legend, table legend, main text, or Methods section).

n/a	Confirmed	
☐	☒	The exact sample size (n) for each experimental group/condition, given as a discrete number and unit of measurement
☐	☒	An indication of whether measurements were taken from distinct samples or whether the same sample was measured repeatedly
☒	☐	The statistical test(s) used AND whether they are one- or two-sided<br>*Only common tests should be described solely by name; describe more complex techniques in the Methods section.*
☒	☐	A description of all covariates tested
☒	☐	A description of any assumptions or corrections, such as tests of normality and adjustment for multiple comparisons
☐	☒	A full description of the statistics including central tendency (e.g. means) or other basic estimates (e.g. regression coefficient) AND variation (e.g. standard deviation) or associated estimates of uncertainty (e.g. confidence intervals)
☒	☐	For null hypothesis testing, the test statistic (e.g. F, t, r) with confidence intervals, effect sizes, degrees of freedom and P value noted<br>*Give P values as exact values whenever suitable.*
☒	☐	For Bayesian analysis, information on the choice of priors and Markov chain Monte Carlo settings
☒	☐	For hierarchical and complex designs, identification of the appropriate level for tests and full reporting of outcomes
☒	☐	Estimates of effect sizes (e.g. Cohen's d, Pearson's r), indicating how they were calculated
☐	☒	Clearly defined error bars<br>*State explicitly what error bars represent (e.g. SD, SE, CI)*

Our web collection on statistics for biologists may be useful.

Software and code

Policy information about availability of computer code

Data collection	Unicorn 7.1<br>BLItz Pro 1.1.0.31<br>Imagequant Il 8.1<br>NIS 4.12.01.64bit<br>Zetaziser 7.12
Data analysis	PRISM 5.0<br>PyMol 2.2.0_0<br>Imagequant Il 8.1<br>Swiss Model (https://swissmodel.expasy.org/)<br>PISA (http://www.ebi.ac.uk/pdbe/pisa/)<br>Excel 2013<br>HADDOCK 2.2 (https://haddock.science.uu.nl/services/HADDOCK2.2/)<br>FIJI/imageJ<br>"MicrobeJ" FIJI/imageJ plugin<br>Zetaziser 7.12

For manuscripts utilizing custom algorithms or software that are central to the research but not yet described in published literature, software must be made available to editors/reviewers upon request. We strongly encourage code deposition in a community repository (e.g. GitHub). See the Nature Research guidelines for submitting code & software for further information.

Data

Policy information about availability of data

All manuscripts must include a data availability statement. This statement should provide the following information, where applicable:

- Accession codes, unique identifiers, or web links for publicly available datasets
- A list of figures that have associated raw data
- A description of any restrictions on data availability

All relevant data are reported in the main text or Supplementary Information. The source data underlying Figure 1C, 3A, 3B, 3C, 4A-B, 5B-D, 7 and Supplementary Figure 4B, 5D, 6B-D, 7-B, and 9 are provided as a Source Data file. Any additional data relevant to this manuscript are available from the authors upon request.

Field-specific reporting

Please select the best fit for your research. If you are not sure, read the appropriate sections before making your selection.

☒ Life sciences ☐ Behavioural & social sciences ☐ Ecological, evolutionary & environmental sciences

For a reference copy of the document with all sections, see nature.com/authors/policies/ReportingSummary-flat.pdf

Life sciences study design

All studies must disclose on these points even when the disclosure is negative.

Sample size	No statistical methods were used to determine sample sizes. Data were collected over multiple days from clonal population, and analyzed.
Data exclusions	No data were excluded from the analysis
Replication	All attempts at replications were successfull. The number of replicates is indicated in the corresponding figure legend and/or the corresponding methods section, each replicates were performed with independant biological samples. Statistical analyses are provided, or a representative experiment is shown.
Randomization	All experiments were performed with clonal populations
Blinding	No blinding was performed as the acquisition and analysis methods required human intervention

Reporting for specific materials, systems and methods

Materials & experimental systems

n/a	Involved in the study	
☒	☐	Unique biological materials
☐	☒	Antibodies
☒	☐	Eukaryotic cell lines
☒	☐	Palaeontology
☒	☐	Animals and other organisms
☒	☐	Human research participants

Methods

n/a	Involved in the study	
☒	☐	ChIP-seq
☒	☐	Flow cytometry
☒	☐	MRI-based neuroimaging

Antibodies

Antibodies used	antibody Penta-His-HRP conjugated (Qiagen, Cat# 34460, Lot# 154045877, dilution 1/10000.<br>anti body Cya A (3D1) mouse monoclonal antibody (SANTA CRUZ BIOTECHNOLOGY, INC. Cat# sc-13582, lot#F2419, dilution 1/5000.<br>Anti-Mouse IgG (whole molecule)–Peroxidase antibodya (Sigma aldrich, Cat#4416, Lot#slbw4917,dilution 1/10 000)
Validation	Antibodies were validated in western-blots with samples expressing tagged and untagged protein
